# Early Detection Methods for Silicosis in Australia and Internationally: A Review of the Literature

**DOI:** 10.3390/ijerph18158123

**Published:** 2021-07-31

**Authors:** Emma K. Austin, Carole James, John Tessier

**Affiliations:** 1 Centre for Resources Health and Safety, College of Health, Medicine and Wellbeing, University of Newcastle, Callaghan, NSW 2308, Australia; Carole.James@newcastle.edu.au; 2 School of Health Sciences, College of Health, Medicine and Wellbeing, University of Newcastle, Callaghan, NSW 2308, Australia; John.Tessier@newcastle.edu.au

**Keywords:** silicosis, pneumoconiosis, early detection, respiratory surveillance

## Abstract

Pneumoconiosis, or occupational lung disease, is one of the world’s most prevalent work-related diseases. Silicosis, a type of pneumoconiosis, is caused by inhaling respirable crystalline silica (RCS) dust. Although silicosis can be fatal, it is completely preventable. Hundreds of thousands of workers globally are at risk of being exposed to RCS at the workplace from various activities in many industries. Currently, in Australia and internationally, there are a range of methods used for the respiratory surveillance of workers exposed to RCS. These methods include health and exposure questionnaires, spirometry, chest X-rays, and HRCT. However, these methods predominantly do not detect the disease until it has significantly progressed. For this reason, there is a growing body of research investigating early detection methods for silicosis, particularly biomarkers. This literature review summarises the research to date on early detection methods for silicosis and makes recommendations for future work in this area. Findings from this review conclude that there is a critical need for an early detection method for silicosis, however, further laboratory- and field-based research is required.

## 1. Introduction

Silicosis is an incurable, sometimes fatal, but completely preventable lung disease caused by exposure to respirable crystalline silica (RCS). Worldwide, thousands of workers in a range of industries are at risk of developing silicosis. Early detection of silicosis is vital to identify the disease at a pre-clinical stage to allow interventions that improve outcomes for workers, while investigating inadequacies in workplace control practices. Currently, international respiratory surveillance includes health and exposure questionnaires, spirometry, X-rays, and high-resolution computed tomography (HRCT). However, some of these techniques are unable to detect silicosis at an early stage. This review investigates current respiratory surveillance for silicosis and explores potential opportunities for alternative early detection methods, particularly biomarkers and exhaled breath condensate (EBC).

Pneumoconioses are a group of non-malignant parenchymal (interstitial) lung diseases caused by inhaling dust particles [1,2]. Worldwide, one of the most common work-related injuries is pneumoconiosis, specifically caused by exposure to RCS [3]. Indeed, in China, pneumoconiosis is the most prevalent occupational disease [4]. Recent years have seen a resurgence of certain types of pneumoconiosis, particularly in the United States [5] and Australia [6]. 

The three primary types of pneumoconiosis are asbestosis, coal workers’ pneumoconiosis (CWP), and silicosis [6]. Silicosis is a fibrotic lung disease caused by inhaling RCS [7]. For both developed and developing countries, silicosis is a major cause of mortality and morbidity [8]. Silicosis is highly prevalent in low- and middle-income countries, although the true extent is likely underreported due to poor respiratory surveillance [7]. 

Australia has recently experienced a surge in silicosis cases as a result of growth in the manufactured stone industry. In 2019, there were an estimated 350 cases of silicosis in Australia, with 100 cases identified between September and December [9]. Given that over 500,000 Australians are exposed to RCS in the workplace annually [10], silicosis has the potential for huge socioeconomic impact. In addition to silicosis, in 2015, the Queensland government received its first report of CWP in over 30 years [6], prompting the Queensland Government Department of Natural Resources, Mines and Energy (DNRME) to conduct a review of the health assessment performed under the Queensland Coal Mine Workers’ Health Scheme [11].

Industries where occupational exposure to RCS is prominent include manufactured stone, stone masons, coal mining, denim blasting, dental technicians, and other various trades [12]. Table 1 demonstrates the broad range of workplaces where RCS exposure can occur. In addition, exposure is also possible via background environmental conditions [13] and volcanic eruptions [14].

Worldwide, silica (silicon dioxide) is a naturally occurring and abundant mineral, forming the major element of most rocks and soils [7,17,18]. There are non-crystalline and crystalline forms of silicon dioxide, with only crystalline forms causing pneumoconiosis. Silica dust is generated in the workplace in a range of industries. Mechanical processes in the workplace, such as sawing, crushing, drilling, polishing, cutting, or grinding of natural stone or manufactured products, produce the harmful dust. Respirable particles are dust particles that are so small they are not visible [17]. In addition to silicosis, RCS is associated with a number of diseases, including lung cancer, chronic obstructive pulmonary disease (COPD), tuberculosis, scleroderma, rheumatoid arthritis, autoimmune diseases (AIDs), and chronic kidney disease [19,20]. Some patients with silicosis are susceptible to also developing tuberculosis (silicotuberculosis) [21].

The symptoms of silicosis differ according to the stage and severity of the disease. Simple silicosis may be asymptomatic and incidentally diagnosed during routine respiratory surveillance. The most commonly recognised form of the disease is chronic silicosis, which usually develops after exposure to low concentrations of silica dust for 10 or more years [8]. Symptoms of chronic silicosis may include cough and shortness of breath. Accelerated silicosis shares some clinical features with chronic silicosis, although it often progresses more rapidly and develops 5–10 years after initial exposure [15]. Acute silicosis is rare and develops after exposure to high concentrations of silica dust for a period of a few weeks to five years [7]. Symptoms of acute silicosis include dyspnoea, dry cough, fever, fatigue, and weight loss, with respiratory failure and death often occurring within a few months [7]. For all three forms of silicosis, the rate of development is dependent on the surface characteristics of the RCS particles and the intensity and duration of exposure [22].

Respiratory surveillance (also referred to as occupational lung disease screening) in Australia and overseas has common elements, although differences do occur due to available technology and the cost of surveillance. Exposure history and respiratory symptom questionnaires constitute the first step in respiratory health surveillance. Spirometry is a second commonly used method. Together with questionnaires and spirometry, medical imaging—commonly radiography (X-ray)—is used for the diagnosis, surveillance, and screening of occupational lung disease. In addition, various HRCT protocols are a final step in the surveillance process. 

Pneumoconiosis appears as small dot-like opacities on chest X-rays and HRCT, with the shape, size, and quantity of these opacities graded to represent the severity of the disease [6]. For X-rays, this grading is typically conducted using the International Labour Office (ILO) Classification System [23] and, depending on the organisational/regulatory requirements, may be interpreted by one or multiple qualified B readers [15].

Specifically, the US follows these standard methods of respiratory surveillance, and there appears to have been little change in this for some time, although some new monitoring techniques continue to be developed [24]. NIOSH [24] outline occupational respiratory surveillance in the US to include questionnaires, radiography, spirometry, and biomarkers. Surveillance has two phases: (i) an initial medical examination that includes history, physical examination, respiratory and cardiovascular examinations, chest X-ray, and pulmonary function testing (FVC and FEV (1 s)); and (ii) periodic medical examinations on an annual basis [24]. These methods, together with sputum cytology and tuberculin skin tests, are identified as the specific medical tests and examinations for the Occupational Safety and Health Administration (OSHA) regulated substances [25]. As identified by this review, NIOSH also recognises that an optimal method for the early detection of pneumoconiosis is yet to be developed.

Following the unexpected reporting in 2015 of the first case of CWP in over 30 years in Australia, there has been a renewal of the Coal Mine Workers’ Health Scheme [6]. Subsequently, the highest number of cases of CMDLD ever diagnosed in Queensland, Australia has been recorded [6]. This resurgence highlights the need for regular respiratory surveillance with a high level of sensitivity.

Most silicosis cases are not diagnosed at an early stage, as the initial phase of the disease is typically asymptomatic [21] and is often undetectable with spirometry and X-ray. Specifically, silicosis can present a diagnostic challenge due to its radiological resemblance and clinical overlap with sarcoidosis, pulmonary tuberculosis, and neoplastic lesions [26]. In addition, barriers to early diagnosis include a lack of a suitable biomarker, poor health-seeking behavior, insufficient occupational healthcare services at workplaces, particularly in developing countries, and unorganised sectors [21]. Impediments to early diagnosis also include a lack of education and understanding of the level of risk associated with RCS and the limitations of the current methods of initial medical screening (i.e., spirometry and X-ray). In Australia specifically, cultural barriers of a predominantly young, migrant workforce, the growth of the manufactured stone industry, and a historical lack of regulation are suggested to have contributed to a resurgence of silicosis in some populations. Surveillance is needed long-term, even after retirement and cessation of exposure, due to the long latency of silicosis. 

Compounding the issue of exposure to RCS is that X-ray and HRCT present the concern of giving a regular radiation dose to workers. For example, in NSW, workers need to be scanned every year for their whole career. Regulators have a duty of care not to expose workers to an ongoing, annual dose of radiation, although it may be argued that the level of radiation is incidental, and must be weighed against the opportunity for a more sensitive test that reliably detects disease [19].

Despite efforts to establish and maintain best practices, respiratory surveillance continues to be a disparate process [27]. Standardisation of the process is required in order to protect workers exposed to RCS.

Despite the acknowledgment that improved detection methods other than spirometry, X-ray, and HRCT are needed, knowledge gaps remain around alternatives. Although research has been conducted into EBC and biomarkers as methods for detecting silicosis, these techniques have not been validated, and remain at an investigative stage. In addition, there is inconclusive evidence as to which biomarker(s) most effectively capture silicosis. 

The literature review was directed by the following research questions:

- What methods are currently used in respiratory surveillance for occupational lung disease? Have they been validated?
- What alternative methods exist or are under investigation, and what evidence is there for the effectiveness of these methods?
- Is there evidence to support conducting a prospective cohort study to test the validity of alternative methods of early detection of silicosis?

The overarching objective of this review is to inform changes to respiratory surveillance with the global goal to reduce the prevalence of silicosis and improve the prognosis of workers who develop silicosis. Although the search did return a large number of studies that investigated treatments, including murine experiments and investigations of DNA, treatments for silicosis are not a focus of this review.

## 2. Materials and Methods

The review involved three separate search strategies: a scoping literature review of peer-reviewed articles, a search of the grey literature, and a search of websites and online material. In addition, the research team consulted with leading academics and regulatory professionals in Australia and overseas to gain insights into the current prevalence of silicosis and screening methods.

### 2.1. Scoping Review

The scoping review was conducted according to the PRISMA-ScR framework. PRISMA-ScR is a systematic approach to assist with mapping evidence on a topic and identifying the main concepts, theories, and knowledge gaps relevant to that topic [28,29].

An initial browsing search of the online database MEDLINE was completed to familiarise the researchers with the key search terms. The scoping review search was conducted in the online library databases Scopus, Embase, and CINAHL. Using the keyword search function, search terms were: “silicosis” or “pneumoconiosis” or “black lung” or “respiratory fibrosis” or “dust disease”, together with “monitor” or “early detect” or “mass screening” or “screen” (truncation and proximity searching were applied to some terms). The search was limited to articles written in English and published since 2010. Articles were catalogued and screened using the referencing software Endnote and the web-based software platform Covidence, which streamlines the production of reviews. The reference lists of included articles were also searched. The search was conducted in March 2020.

### 2.2. Grey Literature

When searching grey literature, it was necessary to keep the search terms more broad than when searching peer-reviewed journal databases. Google Scholar was searched with the terms “silicosis” and “early detection” and “screening”. The records were presented according to relevancy; the first 200 records were screened, and those that were appropriate were included in the review [30]. The Mednar database was searched with the search term “silicosis”. The first 200 records were screened, and relevant documents were included in the review. Mednar conducts a comprehensive search across medical societies, the National Institute of Health resources, US government websites, and patents. The OpenTrials database was searched to find clinical trials specifically relating to silicosis and early detection methods.

### 2.3. Websites, Industry, Government, and Regulators

When searching websites, it is optimal to maintain generic and overarching search terms, as the search relies on the website’s own search engine. Websites searched included regulatory bodies, industry organisations, and government websites in Australia and internationally.

## 3. Results

**Figure 1:**
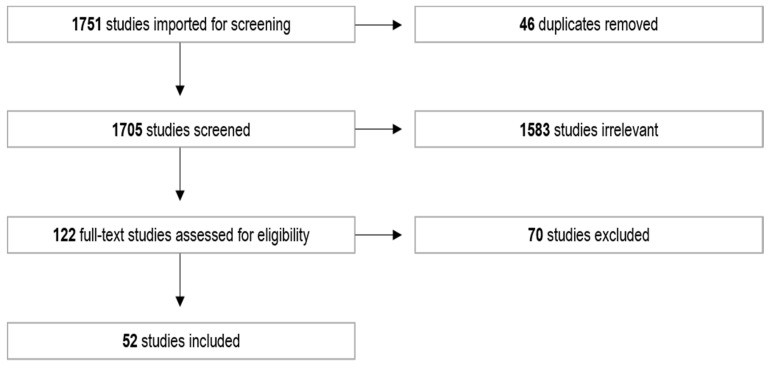
PRISMA flow chart showing the screening process for the scoping review.

The findings from the three search methods are synthesised below. The database search for the review returned 1751 articles. After 46 duplicates were removed, the titles and abstracts of 1705 articles were screened for relevance, of which 122 progressed to full-text screening. This final screening process determined that 52 articles were eligible for the final scoping review. Figure 1 shows the screening process for the scoping review. The final 52 articles included in the scoping review are summarised in Table 2.

In addition, following the search methods outlined above, there were 19 grey literature sources screened and included in the final review; these are summarised in Table 3.

**Figure 2:**
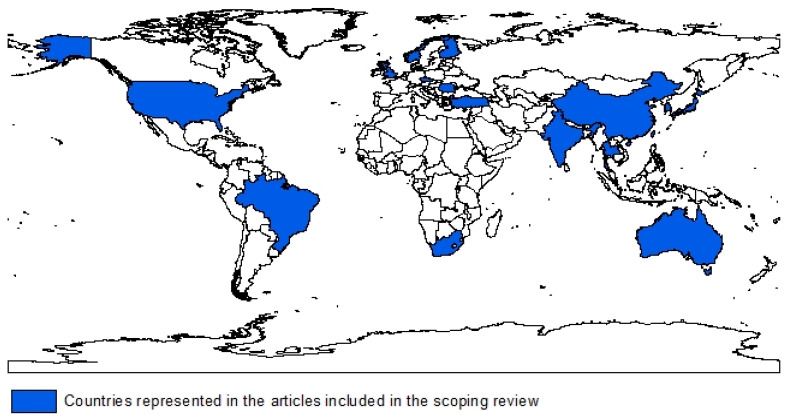
Global incidence of silicosis in the journal articles included in the scoping review.

The countries represented by the peer-reviewed literature included in the scoping review are shown in Figure 2.

The worldwide occurrence of silicosis was demonstrated in the spatial distribution of the studies included. This was not necessarily an exhaustive list of all countries that had incidences of silicosis.

Many studies included in this review reported a high incidence of smoking among participants. This confounder makes it difficult to isolate the impacts of silica dust exposure from the damage caused by smoking. It is common practice for cessation of smoking programs to be promoted at screening appointments and to participants in silicosis research studies.

### Clinical Trials

OpenTrials returned 44 entries when we searched for “silicosis” and “pneumoconiosis”, however, these trials either had no results available or were testing drugs for treatment. It appears from the search conducted for this review that clinical trials investigating early detection methods for silicosis are rare.

## 4. Discussion

This review was guided by the overarching aim to inform changes in respiratory surveillance with the global goal to reduce the prevalence of silicosis and improve the prognosis of workers who develop silicosis. Some articles included in the scoping review focus more broadly on pneumoconiosis in general or other types of pneumoconiosis, such as CWP. The methods investigated in these articles are pertinent to respiratory surveillance for silicosis. Different surveillance methods were identified, including spirometry, imaging, and HRCT, and these are discussed in more detail below.

### 4.1. Spirometry

Spirometry is a type of pulmonary function test. Spirometry is currently used for diagnosing the risk of damage, identifying lung disease, monitoring workers exposed to particulate matter, and to evaluate therapeutic interventions [35]. Although spirometry has been used as the first-choice method to evaluate pulmonary alterations in workers exposed to particulate matter, spirometry has limited sensitivity when detecting abnormalities before extensive damage occurs [35]. In addition, there are different standards for the procedure itself, for example, in Australia, the test must be performed for coal mine workers by practitioners with a particular qualification [77], but this is not required in other occupations. 

Spirometry, or some form of pulmonary function test, was used in many of the studies in the scoping review. In these investigations, spirometry was always accompanied by health and exposure questionnaires and, in most cases, by additional surveillance methods, such as chest X-ray or HRCT [20,45,61,62,66]. Spirometry can contribute to the diagnosis and monitoring of pneumoconiosis, and specifically, Trakultaweesuk et al. [66] found that spirometry, using a mean decline in FEV_1_ of 272.0 ± 155.5, was a good parameter for the respiratory surveillance of silica-exposed workers. It is important to note that spirometry and questionnaires alone are not able to diagnose the difference between silicosis and COPD. Despite the widespread use of spirometry, the practical implications and inconsistencies in performing the test must be considered [77].

### 4.2. Imaging

Respiratory surveillance routinely incorporates imaging, including chest radiography (X-ray) and/or HRCT. Globally, it is typical for chest X-rays to be assessed according to the International Labour Organisation (ILO) Classification System [78]. In addition, many jurisdictions have the requirement for an NIOSH B Reader to assess the chest X-ray, a certification granted to physicians who demonstrate proficiency in the classification of chest X-rays for pneumoconioses using the ILO Classification System [15].

It has been identified that chest X-rays are failing to reliably detect occupational lung disease [19,79]. For example, in a cohort of workers from Queensland, 43% had chest X-rays classified as normal using the ILO Classification System, however, the disease was visible on HRCT [19]. Non-occupational lung disease is now diagnosed using CT, and it is recommended that HRCT also replace chest X-ray for the diagnosis of occupational lung disease due to CT’s higher sensitivity to detect early disease and greater accuracy in characterising patterns of disease [19,80,81]. The Royal Australian and New Zealand College of Radiologists [19] strongly recommend CT as the primary imaging modality to be used for respiratory surveillance in exposed workers. This recommendation is supported by Kahraman et al. [46], and the references therein. 

Specifically, HRCT has an enhanced capacity to detect pneumoconiosis compared to chest X-ray due to the increased sensitivity provided by the finer spatial resolution and 3D nature of HRCT [6]. While Larici et al. [48] concluded that HRCT is the optimal modality of imaging, Şener et al. [63] resolved that, although HRCT had a higher rate of detection in the early stages, the cost, radiation exposure, accessibility, and lack of ability to evaluate pulmonary functions did not support the introduction of routine use in this setting. 

In some jurisdictions, in this case Korea, analog radiography persists as the standard for respiratory surveillance. Lee and Choi [50] concluded that soft images from a flat-panel detector of digital radiography provide more accurate and reliable results in pneumoconiosis classification and diagnosis than analog radiographs, and concluded that, in the circumstance where HRCT is not available, digital radiograph is preferred. Conflictingly, [69] found digital and analog radiography to be equivalent. 

There is a body of work that investigates the automatic classification of chest X-rays [57,64,72,73,75,76]. In some locations, there is a lack of expertise in the diagnosis of occupational lung disease, and it appears that these technologies have the capacity to assist by automatically detecting abnormalities in chest X-rays.

As stated previously, detection in the early stages of silicosis has challenges. McBean et al. [6] described radiologists as being at the frontline in occupational lung screening and that they must be aware of the imaging spectrum.

### 4.3. Biomarkers

There is a growing body of epidemiological research that focuses on validating biomarkers by assessing their ability to indicate exposure, effect, disease, or susceptibility [82]. When used in health surveillance, biomarkers can be indicators of hazard, exposure, disease, and population risk [83]. The overarching goal of using biomarkers is to provide insight into the pathogenesis of silicosis and the biological mechanisms that underpin its progression. This review identified a number of studies that aimed to validate particular biomarkers as indicators of silicosis [4,36,37,42,49,61,67]. The grey literature search returned several Chinese articles about biomarkers that could not be accessed.

Thakkar et al. [84] identified that existing studies that consider biomarkers have been conducted with cross-sectional methods within a group population over a short time period. These findings give statistical and probabilistic results in terms of an individual subject. However, what is needed is an observation of biomarkers over time, i.e., a longitudinal cohort study is essential. A study of this design would have prognostic value and contribute to workers adopting preventive strategies, while also reducing individual cases of silicosis [84]. 

Many studies test for biomarkers of oxidative stress, an imbalance in the body between the production of free radicals and the antioxidant defense [65]. Oxidative stress can lead to damage in biological tissue as a result of an imbalance between oxidants and antioxidants [82]. Metals found in mine dust have the potential to induce oxidative stress, which can cause harmful effects to the human body [43]. The ability of a chemical to exert biological effects dictates the capacity to generate oxidative stress [43]. Oxidative stress has been identified as strongly related to the severity of silicosis [12]. However, the parameters of oxidative stress that represent silicosis remain invalidated.

There are many avenues of biomarkers that require further investigation. It has been identified that, in the search for biomarkers for pneumoconiosis, there is a need to investigate biomarkers that play important roles in screening, diagnosis [74], and disease progression [85]. In addition, Schulte [83] notes the need to justify the cost and difficulty in obtaining samples. Pandey and Agarwal [85] emphasise the need for a cohort and longitudinal study of the potential biomarkers in vulnerable groups. 

A large number of biomarkers with the potential to detect lung disease were investigated in the literature summarised in this review, including (but not limited to): Club/Clara cell protein 16 (CC16) [21]; serum HO-1 [61]; IL6 [53]; TNFα, IL6, and IL8 [47]; and Npnt [49]. It was not possible to determine a single biomarker with the most potential. Indeed, the need for research to identify biomarkers that provide insight into the pathogenesis of silicosis and the biological mechanisms that underpin its progression was abundantly clear. 

### 4.4. Exhaled Biomarkers

Exhaled breath condensate (EBC) can be used to assess the respiratory health of pneumotoxic-exposed workers, as it quantifies lung tissue dose and the consequent pulmonary effects [39]. EBC is obtained by collecting exhaled cooled breath, which is analysed for volatile and non-volatile macromolecules [86]. The range of biomarkers that have been explored when investigating pneumoconiosis, including oxidative stress and inflammatory-derived biomarkers, suggests that EBC analysis may contribute to understanding the pathogenesis of the airways of exposed workers [39]. EBC analysis, as a method of studying pulmonary biomarkers of exposure, effect, and susceptibility in the workplace, proves to be one of the most promising methods currently available [39]. In particular, due to its non-invasive collection method, it is highly suitable to be applied in field studies and longitudinal assessments [39]. 

Although not commonly used, findings support the suggestion that EBC can contribute to an improved understanding of the pathogenesis of silicosis [59,60]. Indeed, when compared to plasma and urine, markers in EBC appeared to be the most useful method for detecting pneumoconiosis [60]. Leese et al. [87] demonstrated that crystalline silica particles can be detected in the EBC of exposed workers, however, there were limitations due to the volume of the sample produced.

The measurement of exhaled NO and volatile organic compounds is considered to be an inexpensive, safe, and easy-to-perform test that can be used to assess peripheral lung inflammation, and could potentially play a role in the diagnosis and follow-up of fibrosing lung disorders [62,71]. However, further research is needed that includes follow-up testing and investigating different levels of exposure [62].

EBC is non-invasive and highly accurate, making it an attractive option for the early detection of silicosis. Again, there is a growing body of research investigating a number of EBC options, and the need for further study is acknowledged [62,71,87]. Indeed, Corradi et al. [39] recognised the substantial limitations that currently exist, preventing its use as a routine method of screening in the workplace. Specifically, they identified the need for further development in the area of standardising EBC collection, data reporting, and validation of biomarkers.

### 4.5. Summary of Methods

This review identified the need to standandarise the process of respiratory surveillance. In addition, X-ray was determined as not sufficient in detecting silicosis, while spirometry is subject to the skill and experience of the practitioner. HRCT is recognised as the optimal method, however, it is not always available. EBC and biomarkers hold promise, although, at this stage, they are not validated and remain at an investigational stage.

The strengths of this review include the search being conducted beyond peer-reviewed literature to include grey literature and supporting regulatory documentation, as well as the scoping review following the systematic PRISMA-ScR framework. However, it should be noted there are some limitations, such as including only papers published in English, and the fact that only those published since 2010 were included. 

Based on the findings presented here, a number of recommendations were formulated. Firstly, there is a need for further lab- and field-based studies that monitor a range of biomarkers to successfully identify one or more biomarkers that conclusively provide insight into the pathogenesis of silicosis and the biological mechanisms that underpin its progression. Second, any future empirical studies that attempt to validate the use of biomarkers or EBC as an early detection method for silicosis must also include standard surveillance methods as a point of comparison, i.e., spirometry, X-ray, and HRCT. Lastly, future empirical studies should include a diversity of participants to allow examination of a range of scenarios, for example, diagnosed silicosis patients at different stages of the disease, exposed workers (with no previous diagnosis of silicosis, COPD, TB, fibrosis, etc.) with a varying number of years of occupational exposure, and healthy unexposed controls with no previous diagnosis of silicosis, COPD, TB, fibrosis, etc. 

## 5. Conclusions

Silicosis is a debilitating and sometimes fatal disease, yet it is totally preventable. Caused by exposure to RCS, hundreds of thousands of workers worldwide are at risk of developing silicosis. The global prevalence of silicosis (and other pneumoconioses) warrants further investigation into methods for detection in the early stage of the disease. While spirometry, X-ray, and HRCT can play important roles in respiratory surveillance, there is opportunity for new methods, such as biomarkers and EBC, to become routine methods of surveillance. Any future effort to research into early detection methods for respiratory surveillance should focus on providing insight into the pathogenesis of silicosis and the biological mechanisms that underpin its progression. These efforts should include longitudinal analysis of at-risk populations.

## Figures and Tables

**Table 1:** Common operations or tasks that involve exposure to free crystalline silica [7,15,16].

Activity	Industries or Occupational Activities
Drilling	Construction, quarrying and related milling, mining and related milling, tunneling
Breaking and crushing	Construction, quarrying and related milling, mining and related milling, tunneling
Cutting	Arts, crafts, and sculpture, jewelry, construction, quarrying and related milling, grindstone production
Abrasive blasting and sand blasting	Boiler scaling, production of dental material, metal products, automobile repair (removal of paint and rust), arts, crafts, and sculpture, shipbuilding and repair, foundries, construction, quarrying and related milling, production of denim jeans, tombstone production
Grinding	Arts, crafts, and sculpture, jewelry, construction, quarrying and related milling
Sanding	Automobile repair (removal of paint and rust), construction
Excavation and digging	Agriculture, construction, quarrying and related milling, mining and related milling, tunneling
Hammering	Boiler scaling, construction
Casting and moulding	Jewelry, foundries, ceramics
Furnace installation and repair (refractory materials)	Iron and steel mills, foundries, glass
Cleaning (dry sweeping and brushing, pressurised air blowing)	Construction, arts, crafts, and sculpture, jewelry
Polishing and buffing	Production of dental material, arts, crafts, and sculpture, jewelry

**Table 2:** Extraction table of the 52 articles included in the scoping review.

Author (Year)	Objective/s	Type of Study (Cross-Sectional, Clinical Trial, Longitudinal, Review)	Population/Exposure (Years)	Population Size	Gender	Age (Years)	Respiratory Surveillance Method/S Used	Outcomes	Location
Aggarwal [31]	Investigate total lactate dehydrogenase (LDH) activity in blood samples as a non-invasive method to measure silica-induced toxicity. First study to estimate LDH activity in blood cells of silica-exposed agate workers and controls. Proposes LDH activity as a diagnostic tool for early silica exposure-induced cytotoxicity.	Cross-sectional	Silica-exposed agate workers	Exposed workers: 35 Control subjects: 27	Exposed workers: 21 male	Exposed workers: 42 ± 11	Blood sample	Blood cells: LDH activity significantly higher (~10×) in control subjects, suggesting the blood cells of exposed workers may have been damaged directly or indirectly by silica exposure	India
			Exposure: 16 ± 8		Control subjects:27 male	Control subjects: 31 ± 9		Blood plasma: LDH activity is higher (~25×) in exposed workers, suggesting that silica exposure may have induced cellular and tissue injuries, with more extracellular LDH enzyme released into blood plasma	
Alexopoulos et al. [32]	Compare cellular profiles of asbestosis-exposed workers using induced sputum (IS) and bronchoalveolar lavage fluid (BALF) to test the usefulness of IS in monitoring workers over an extended period. Validate screening tool for biological	Cross-sectional	Workers at a car brakes and clutches factory that uses chrysotile asbestos	Workers: 39	Workers: 24 male	37–53	Questionnaire, Bronchoscopy, Induced sputum	Findings detected significant correlations between IS and BALF cellular profiles. This indicates that IS sampling, a less invasive and expensive method, may provide useful insights both for inhalation of dusts and inflammatory processes in the lung.	Romania
			Exposure: >15						
			Absence of diagnosis of pneumoconiosis						
Aslam et al. [33]	Develop fluorescence-based analysis tool to monitor in real-time the LOX enzyme activities in vitro and in vivo of patients with fibrogenesis. These powerful tools are a simple and effective method of monitoring.	Cross-sectional	Human and asinine ex vivo tissue models	Human lung samples: 17	N/A	Human lung samples: 55–81	Lung samples from carcinoma resections, Biopsy, ex vivo asinine lung samples	Successful design of an activity-based fluorescent probe that quantifies in real-time the LOXF activity in fibrogenic conditions. This probe has the potential to image real-time LOXF activity within the lungs of patients.	United Kingdom
				Number not stated		Asinine lungs: aged			
Brilland et al. [20]	Analyse the impact of crystalline silica on T cell phenotype and regulatory T cells (Tregs) frequency.	Prospective cohort	Workers with moderate to high levels of exposure	Exposed workers: 55	Males	Exposed workers: 41.30 ± 6.52	Clinical examination, chest X-ray, pulmonary function test, blood sampling	Alterations of the T cell compartment can be detected early during the course of crystalline silica exposure, hence preceding the development of silicosis or autoimmune diseases.	France
				Two cohorts of HC; group 1 = 42 and group 2 = 45		Control group 1: 41.67 ± 12.59 Control group 2: 42.06 ± 8.89			
Chao et al. [34]	Investigate the mechanisms underlying endothelial-mesenchymal transition (EndMT)	Lab based	N/A	N/A	N/A	N/A	N/A—lab based	Findings suggest MCPIP1-induced EndMT in endothelial cells plays an important role in the development of silicosis.	China
Chao et al. [35]	Investigate if cardiopulmonary exercise testing may be better than spirometry when used to detect early signs of damage caused by occupational exposure to particulate matter. First study to focus on early detection in asymptomatic participants.	Cross-sectional	Male workers from the Epidemiology and Human Movement Study (EPIMOV). Completed a validated occupational respiratory questionnaire to determine occupational exposure to particulate matter.	Exposed = 52	Male only	>18		Male workers exhibited ventilatory alterations during exercise, even with normal pulmonary function at rest. Findings suggest ∆VT/∆InVE may be the most appropriate variable from the CPET to differentiate workers with incipient ventilatory changes. CPET may be useful in the prevention of occupational respiratory diseases.	Brazil
				Control = 83					
Chu et al. [4]	To systematically evaluate genetic variants that were associated with pneumoconiosis susceptibility.	Three-stage case-control	Exposed coal and metalliferous underground miners	Cases: 202	N/A	N/A	Physical examination, radiograph, genome samples	Identified a genome-wide significant association and two additional replicated associations for pneumoconiosis susceptibility in Han Chinese.	China
				Control: 198					
Chu et al. [36]	Identify miRNA as potential diagnostic biomarkers for silica-related pulmonary fibrosis.	Three-stage case-control	See Table 1 in article	Cases: 67	See Table 1 in article	See Table 1 in article	Blood samples	miRNA-4508 may be a potential diagnostic marker for silica-related pulmonary fibrosis, and a functional variant of miRNA-4508 may affect susceptibility.	China
				Controls: 67					
Chu et al. [37]	Investigate the causal variants of chromosome 12q15 in silicosis susceptibility.	Case-control	Case: 24.58 ± 7.00	Cases:177	Case: Male 89.27%	Case: 67.70 ± 8.49	Blood samples	A variant of the carboxypeptidase M (CPM) gene may increase silicosis susceptibility. Provide insight into the aetiology and biological mechanisms of silicosis. Assist in identification of high-risk individuals with occupational silica-exposure.	China
			Control: 24.05 ± 5.69	Healthy controls: 204	Control: 84.80%	Control: 60.26 ± 6.35			
Codorean et al. [38]	Perform exploratory study on peripheral whole-blood to analyse early effects of exposure in coal fired power plants.	Cross-sectional	Three groups: 10 years; 20 years; control	N/A	N/A	N/A	Blood samples	This method is non-invasive and rapid and could be a useful tool in identifying early hazard before it is diagnosed clinically.	Romania
Corradi et al. [39]	Review EBC studies that investigate exposure and effect biomarkers in lung disease, particularly toxic metals	Review	N/A	N/A	N/A	N/A	Exhaled breath condensate (EBC)	Exhaled breath biomarkers have been shown to be capable of detecting and monitoring diseases of the respiratory system.	N/A
Cox and Lynch [40]	Provide review of recent developments in medical imaging of environmental lunch disease.	Review	N/A	N/A	N/A	N/A	Medical imaging	Medical imaging is useful in the diagnosis, epidemiological study and management of occupational lung disease. Studies that compare HRCT with film-screen radiography found CT was more sensitive.	N/A
Dinescu et al. [41]	Identify correlations between electrocardiographic and echocardiographic changes in patients with silicosis prior to chronic pulmonary heart disease occurring.	Prospective, descriptive, analytical	N/A	Cases: 67	N/A	Cases: 477–8 years	Electrocardiograph, echocardiograph	Values of the right heart echocardiographic parameters at the upper limit of normality are early markers for cardiovascular damage in patients with silicosis.	Romania
				Control: 25					
Doganay et al. [3]	Assess MDCT findings of silicosis in denim sandblasters and define the role of MDCT in the early detection of silicosis.	Cross-sectional	Denim sandblasters	12 male patients admitted to a pulmonary outpatient clinic between April-December 2009.	Male only	19–25 years	CT	Silicosis may cause immediate mortality, especially in young people. MDCT can play an important role in the early detection of silicosis.	Turkey
			1.0–5.2 years			21.2 ± 1.2			
			3.7 ± 1.4						
Ehrlich et al. [42]	Estimate the effect of respirable dust and quartz exposure on spirometric lung function.	Cross-sectional	Black South African gold miners	520 mine workers	Not reported	37–60 years	Questionnaires, X-ray, spirometry	Study demonstrated significant lung function loss attributable to dust exposure, mediated by silicosis, pulmonary TB and/or an independent dust effect.	South Africa
			6.3–34.5 years			Mean = 46.7 years			
			Mean = 21.8 years						
Greabu et al. [43]	Evaluate the relationships between occupational exposure to mine dust and salivary antioxidants, blood uric acid and the possible implications for the causes of diseases caused by exposure.	Cross-sectional	Long-term occupational exposure in non-ferrous metal mines	Exposed workers: 30	Not reported	Exposed: 44.3 (SD) 4.5	Saliva samples	First study to describe saliva and serum parameters involved in antioxidant protection and metabolic regulations in non-ferrous metal miners.	Romania
				Control: 30		Control: 51.3 (SD) 5.6			
Guo et al. [44]	Survey and identify differentially expressed circulating miRNAs by miRNA deep sequencing blood samples from patients at varying stages of CWP.	Case-control	N/A	Cases: 30	N/A	N/A	Blood samples	Demonstrated that expressed circulating miRNAs showed dynamic expression patterns across diseased samples. This suggests these miRNAs may have critical roles in the occurrence and development of CWP.	China
				Controls:10 *n* = 456					
Johnsen et al. [45]	Investigate the relationship between dust exposure and annual change in lung function among Norwegian silicon carbide exposed workers using a quantitative job matrix (JEM) regarding total dust	Longitudinal cohort	Workers in Norwegian silicon carbide plants	See Table S2a,b. Examinations = 1499	N/A	N/A	Questionnaires, spirometry, JEM (dust exposure matrix)	Dust exposure, expressed by quantitative JEM, was found to be associated with an increased yearly decline in FEV1. A dose-response relationship was found.	Norway
Kahraman et al. [46]	Document pulmonary function and prevalence of pneumoconiosis in dental prosthetic technicians	Cross-sectional	Dental prosthetic technicians 16.7 ± 8.4 (4–43)	*n* = 76	Male	32 ± 8, (18–55)	Physical examination, Pulmonary function test, HRCT	First prevalence study in dental prosthetic technicians using HRCT. Pneumoconiosis was detected in 46%, possible because HRCT is able to detect very early changes.	Turkey
Kamaludin et al. [47]	Determine biomarker to be used in diagnosis of occupational airways inflammation from occupational inorganic dust exposure.	Review	N/A	N/A	N/A	N/A	Biomarkers	Three biomarkers were identified.	Malaysia
Larici et al. [48]	Highlight the current role of imaging, describe classic as well as uncommon HRCT patterns helpful in guiding diagnosis.	Review	N/A	N/A	N/A	N/A	HRCT	HRCT is the best imaging modality. Imaging plays a role in diagnosis, surveillance, and prediction.	N/A
Lee et al. [49]	Review the roles of previously identified molecules in silicosis-related lung fibrosis from the literature.	Review	N/A	N/A	N/A	N/A	Biomarkers	Serum Npnt was higher in silicosis patients compared to healthy controls. Serum Npnt seems to play a role in progression of fibrosis with other cytokines, and may therefore be a suitable biomarker.	N/A
Lee and Choi [50]	Evaluate the reliability and validity of soft copy images based on flat-panel detector of digital radiography compared to analog radiographs in pneumoconiosis classification and diagnosis.	Cross-sectional	Retired workers exposed to inorganic dusts	*n* = 349	N/a	62.4 ± 7.8	Digital and analog radiography	Flat-panel detector of digital radiography soft copy images showed more accurate and reliable results in pneumoconiosis classification and diagnosis than analog radiographs.	Korea
			19.8 ± 8.1						
Lee and Choi [51]	Compare digital and analog radiography for screening of pneumoconiosis with respect to radiation dose, image quality, and classification.	Cross-sectional	Exposed to inorganic dust	*n* = 531	Male	61.1 ± 8.3 (43–79)	Digital and analog radiography	Compared to analog radiography, digital radiography provides improved image quality with a significant reduction of up to 23.6% in radiation dose and more accurate pneumoconiosis classification.	Korea
			19.5 ± 8.2 (3–45)						
Lee et al. [52]	Develop an improved set of standard digital images to be used in the recognition and classification of pneumoconiosis.	Cross-sectional	Exposed to inorganic dust	*n* = 531	Male	63.1 ± 7.9 (42–84)	Digital and analog radiography	A set of 120 standard digital images was developed with more various pneumoconiosis findings than the ILO SARS. They can be used for the digital reference images for recognition and classification of pneumoconiosis.	Korea
			19.5 ± 8.2 (3–45)						
Lewis and Fishwick [27]	Identify areas of good practice within respiratory health surveillance and to formulate recommendations for practice	Review	N/A	N/A	N/A	N/A	N/A	Respiratory health surveillance remains relatively disparate and would benefit from standardisation.	N/A
Liu et al. [53]	Study expression changes in inflammation-related genes in peripheral blood of patients with pneumoconiosis and explore the possibility of these genes as biomarkers.	Cross-sectional	N/A	Various populations	Male	Various populations	Blood samples	IL6 was identified as being possibly involved in the development of pneumoconiosis.	China
Mao et al. [54]	Evaluate the applicability of digital radiography.	Cross-sectional	Dust exposed workers	192	Male 95.3%	Mean = 55.7	Film screen and digital radiographs	Findings demonstrate that digital systems are equivalent to traditional film-screen radiography in the recognition and classification of small opacities.	China
McBean et al. [6]	Understand the radiological presentation of individuals diagnosed with coal mine dust lung disease since 2015 in Queensland.	Case series	Individuals identified as having coal mine dust lung disease (CMDLD) since 2015	79	Male	Mean = 58.9 years (range: 35–90)	Questionnaires, X-ray and/or CT, spirometry	First study in over 30 years to investigate the radiological presentation of CMDLD in QLD, and the first ever to incorporate HRCT. Approximately 30% of subjects had advanced disease. Findings of interest included the high burden of opacities observed and the presence of RCS-related features in the majority of subjects.	Australia
			Mean: 26.2 years (range: 6–45)						
Miao et al. [55]	Conduct proteomic profiling for the early stages of silicosis to investigate the pathophysiology and to identify potential candidate proteins for early diagnosis.	Case-control	Dust-exposed workers without silicosis; silicosis patients; Healthy controls	45	N/A	55–64	X-ray, blood sample	A number of proteins involved in silicosis development were identified, with a large number of proteins and peptides being dramatically altered during early development. This may contribute to future work to identify potential biomarkers.	China
Nardi et al. [56]	Evaluate inflammatory and oxidative stress parameters as potential early biomarkers for RCS exposure.	Case-control	CS exposed miners	Workers exposed to CS = 38	Male	Various, see Table 1 in article	Blood sample, anthropometric measurements,	For the first time, this study suggested L-selectin surface protein expression in lymphocytes might be a potential biomarker for monitoring CS toxicity in workers with at least 16 years exposure.	Brazil
				With silicosis = 24					
				Unexposed workers = 30					
Okumura et al. [57]	Investigate the effects of parameters on overall classification performance. Develop enhancement methods to reduce false-positive and false-negative values in a CAD scheme for pneumoconiosis.	Retrospective, cross-sectional	N/A	N/A	N/A	N/A	Chest radiographs	Successfully developed a CAD system using three new enhancement methods for classification of pneumoconiosis chest radiographs.	Japan
Ophir et al. [58]	Screen exposed workers using quantitative biometric monitoring of functional and inflammatory parameters.	Case-control	Artificial stone workers	Exposed workers: 68	Male	Exposed workers: 48.6 ± 11.4	Questionnaires, PFT, induced sputum	Reports first application of XRF technology for quantifying elements in biological samples. PFT were significantly lower for exposed workers. Also IS in exposed workers showed significantly higher neutrophilic inflammation. Particle size in IS of exposed workers was similar to the artificial stone dust.	Israel
			Up to 20 years	Controls: 48		Controls: 38.0 ± 17.1			
Palabiyik et al. [12]	Investigate if occupational silica exposure results in alterations in neopterin levels, tryptophan degradation, and activities of superoxide dismutase and catalase, agents in the antioxidant defense system.	Case-control	Denim sandblasting workers	Silicosis patients: 55	Male	Silicosis patients: 30 ± 1 (21–48)	Questionnaires, PFT, blood samples	Denim sandblasters exposed to silica had increased neopterin levels and tryptophan degradation confirming the possibility of their use as indicators of cellular immune response.	Turkey
			33.6 ± 23.8 (2 to 120) months	Controls: 22		Controls: 36 ± 10 (18–52)			
Pelclová et al. [59]	Evaluate the potential impact of lung fibrosis on the levels of oxidative stress markers in blood and urine of workers exposed to silica.	Case-control	Various occupations with exposure	Asbestos exposed workers: 45	Asbestos exposed workers: 24 male	Asbestos exposed workers: 69.6 ± 2.0	Questionnaires, physical examination, X-ray, CT, blood sample, urine sample, lung function, EBC	8-isoprostane appears to be the optimal oxidative stress marker for respiratory disorders. HNE can be used as a marker for pneumoconiosis. Findings support the suggestion that EBC can contribute to a better understanding of the pathogenesis of silicosis.	Czech Republic
				Silica exposed workers: 37	Silica exposed workers: 36 male	Silica exposed workers: 69.1 ± 2.9			
				Controls: 29	Controls: 20 male	Controls: 67.0 ± 4.6			
Pelclová et al. [60]	Measure multiple markers in the EBC, plasma and urine of exposed workers to determine the possible impact of systemic disease, pharmaceuticals and diet on EBC levels.	Case-control	Various occupations with exposure	Asbestos exposed workers: 45	Asbestos exposed workers: 24 male	Asbestos exposed workers: 69.6 ± 2.0	Questionnaires, physical examination, X-ray, CT, blood sample, urine sample, EBC	Findings suggest that for the detection of pneumoconiosis EBC is the most useful compared to plasma and urine.	Czech Republic
				Silica exposed workers: 37	Silica exposed workers: 36 male	Silica exposed workers: 69.1 ± 2.9			
				Controls: 27	Controls: 18 male	Controls: 66.0 ± 6.9			
Sato et al. [61]	Identify predictive factors of excess decline in FEV1 in patients with chronic silicosis.	Cross-sectional	Exposed workers	*n* = 33	Male	73.5 ± 5.7	Questionnaires, X-ray, spirometry, blood samples	Serum H01 may be a useful marker of lung function decline and disease progression in silicosis patients.	Japan
			21.9 ± 12.3						
Sauni et al. [62]	Investigate responses to silica exposure, by testing the effects of silica dust on exhaled nitric oxide.	Case-control	Exposed workers in prefabrication factories, quarries and stone-cutting industry	Exposed workers: 94	Male	Exposed workers: 60.4 (40–78)	Exhaled NO, blood samples, spirometry	Measurement of nitric oxide concentration, plasma cytokine and adipokine levels appears to offer a novel method of demonstrating the inflammatory effects of silica exposure. Measurement of exhaled NO is safe, easy to perform and inexpensive.	Finland
			31.0 (SD8.1)	Controls: 35		Controls: 62.1 (49–72)			
Şener et al. [63]	Compare the ability of chest X-ray (ILO classification) and HRCT (ICOERD) to make an early diagnosis of pneumoconiosis.	Retrospective, cross-sectional	Various exposed workers diagnosed with pneumoconiosis	83	Male	44.46 ± 11.45	Chest X-rays, CT, PFT	ILO categories and ICOERD grades were significantly correlated. HRCT performed better when detecting pneumoconiosis in an early stage, however not in evaluating pulmonary functions.	Turkey
Sundararajan et al. [64]	Investigate a method to automatically detect pneumoconiosis on the basis of digital chest X-rays.	Cross-sectional	N/A	N/A	N/A	N/A	Chest X-rays	The method successfully allows practitioners to classify normal versus pneumoconiosis patients.	N/A
Syslová et al. [65]	Determine concentration levels of oxidative stress biomarkers in the EBC of patients with pneumoconiosis.	Clinical study	Pneumoconiosis patients with exposure to silica or asbestos for 22 ± 6 years	*n* = 10	Male	Patients: 69 ± 8	EBC,	There was a statistically significant difference in biomarkers’ concentration levels between the pneumoconiosis patients and the control subjects.	Czech Republic
						Control: 67 ± 4			
Trakultaweesuk et al. [66]	Estimate FEV1 decline at one year follow-up among workers with normal or early abnormal ILO classified chest X-rays.	Descriptive, longitudinal	Exposed sandstone workers (median exposure 6.5 years)	*n* = 52	Female (65.4%)	48 ± 8.9 (27–65)	Questionnaire, spirometry, chest X-ray	A significant loss of lung function was found, despite being only a one-year follow-up. Spirometry was found to be effective in monitoring the effect of exposure on sandstone workers.	Thailand
Uygur et al. [67]	Investigate the relationship between platelet indices and CWP.	Case-control	Retired coal miners	Retired workers with CWP: 97	Male	Retired workers with CWP: 61.9 ± 4.8	Questionnaire, blood sample, chest X-ray	Platelet indices may be considered as biomarkers for the progression of pneumoconiosis.	Turkey
			20.5 ± 3.6	Controls: 50		Controls: 62.3 ± 1.9			
Weissman [68]	Provide update on literature relevant to using CT as a tool for preventing occupational respiratory disease.	Review	N/A	N/A	N/A	N/A	N/A	Although HRCT is more sensitive than X-ray there are insufficient data to determine the effectiveness of HRCT in improving individual outcomes. However, if HRCT is used to screen populations, the ICOERD classification has been shown to be an important tool.	N/A
Xing et al. [69]	Compare film-screen radiography and HRCT for the recognition of the profusion of small opacities and to evaluate the role of HRCT in CWP diagnosis.	Cross-sectional	Coal miners	Coal miners with CWP: 96	Male	Coal miners with CWP: 49.01 ± 6.16	Film-screen radiography, HRCT	HRCT was more sensitive than film-screen radiography in recognising the profusion of small opacities. Findings provide evidence of the advantages of HRCT in diagnosis of pneumoconiosis.	China
				Healthy coal miners: 67		Healthy coal miners: 47.12 ± 7.35			
				Controls:37		Controls: 46.67 ± 6.76			
Xue et al. [70]	Investigate and evaluate the diagnostic values of pneumocyte-derived biomarkers in various pneumoconioses.	Case-control	Patients with asbestosis/silicosis and dust exposed workers	Patients with asbestosis: 43	Patients with asbestosis: 19 male	Patients with asbestosis: 68.2 ± 8.6	HRCT, X-ray, blood samples, pulmonary function test	The combination of KL-6, SP-D and MMP-2 may improve the diagnostic sensitivity for asbestosis and silicosis.	China
				Patients with silicosis: 45	Patients with silicosis: 23 male	Patients with silicosis: 65.1 ± 11.3			
				Dust exposed workers: 40	Dust exposed workers: 21 male	Dust exposed workers: 63.1 ± 8.7			
				Controls; 45	Controls; 22 male	Controls; 65.6 ± 11.4			
Yang et al. [71]	Develop a breath test to detect pneumoconiosis using volatile organic compounds generated from lipid peroxidation.	Case-control	Exposed stone workers	Cases:25	Cases: 68.0%	Cases: 60.0 (9.2)	Questionnaires, physical examination, X-ray, pulmonary function test, fractional exhaled nitric oxide test, blood and urine samples.	Analysis of VOCs in breath is a novel respiratory screening method. Three VOCs were identified as constituting a distinct fingerprint in the breath of pneumoconiosis patients, demonstrating exhaled breath could be used in screening.	Taiwan
			Cases: 19.8 (14.5)	Controls:154	Control: 46.1%	Controls: 50.3 (11.8)			
			Controls: 17.6 (13.8)						
Young et al. [72]	Evaluate the use of CAD to diagnose both TB and silicosis in a population with a high burden of both diseases.	Quantitative	N/A	N/A	N/A	N/A	X-ray	Using CAD as a mass screening tool for TB and silicosis shows promise, however current ability to differentiate between the two is limited. The successful use of CAD to streamline the process of detection requires knowledge of the local context.	South Africa
Yu et al. [73]	Establish an automated scheme for CAD of pneumoconiosis in X-rays	Quantitative	N/A	Normal: 300	N/A	N/A	X-ray	Findings show high classification performances. The fully automated scheme developed in this study has a higher accuracy and a more convenient interaction compared to previous methods. Scheme may be helpful to clinicians using CAD for mass chest screening and interpreting and differentiating between normal and pneumoconiosis cases.	China
				Pneumoconiosis: 125					
Zhang et al. [74]	Construct a phage display human antibody library (PDHAL) against pneumoconiosis for the diagnosis and treatment of CWP.	Case-control	N/A	Patients with pneumoconiosis: 25	Male	Coal workers: 37.51 ± 6.75	Blood samples, BALF	A PDHAL against CWP was established and six strong positive clones were selected, sequenced and identified. Protective factors were identified. Serum and antibodies that could be used as potential biomarkers for the diagnosis and treatment of CWP were identified.	China
				Coal workers with CWP: 558		Controls: 36.88 ± 9.39			
				Coal workers without CWP: 309					
				Control: 393					
Zhao et al. [75]	Describe a CAD method to classify pneumoconiosis on HRCT images.	Quantitative	N/A	Subjects:112	N/A	N/A	HRCT	Findings indicate that the method developed could be helpful in classifying pneumoconiosis on HRCT.	Japan
				HRCT scans: 175					
Zhu et al. [76]	Propose a multi-scale opacity detection approach to detect suspected opacities from X-ray	Quantitative	N/A	N/A	N/A	N/A	X-ray	Findings demonstrate the approach to be effective in detecting and recognising silicosis opacity. The approach successfully revealed changes in silicosis pathology and may be adopted as an appropriate tool for automatic silicosis diagnosis.	China

**Table 3:** List of grey literature included in the review.

Author/Organisation	Available at
Alif et al. [1]	https://www.safeworkaustralia.gov.au/doc/occupational-lung-diseases-australia-2006-2019
International Labour Organisation (ILO)	https://www.ilo.org/global/topics/safety-and-health-at-work/areasofwork/occupational-health/WCMS_108548/lang--en/index.htm
Australian and New Zealand Society of Occupational Medicine (ANZSOM)	https://www.anzsom.org.au/
Coal Services NSW	https://www.coalservices.com.au/wp-content/uploads/2019/11/20180625_Order-43_Information-for-employers_updated-Nov2019.pdf
Icare	https://www.icare.nsw.gov.au/news-and-stories/reducing-worker-risks-for-silica
Lung Foundation Australia	https://lungfoundation.com.au/drive-change/
National Institute for Occupational Safety and Health (NIOSH)	https://www.cdc.gov/niosh/topics/silica/default.html https://www.cdc.gov/niosh/topics/surveillance/ORDS/ https://www.cdc.gov/niosh/topics/surveillance/ords/workermedicalmonitoring.html https://www.cdc.gov/niosh/docs/2005-110/nmed0205.html; https://www.cdc.gov/niosh/docs/81-123/default.html
Royal Australian and New Zealand College of Radiologists (RANZCR)	https://www.ranzcr.com/search/silicosis-position-statement
Royal Australian College of Physicians	https://www.racp.edu.au/advocacy/division-faculty-and-chapter-priorities/faculty-of-occupational-environmental-medicine/accelerated-silicosis/faqs
Safe Work Australia	https://www.safeworkaustralia.gov.au/silica
SafeWork NSW	https://www.safework.nsw.gov.au/hazards-a-z/hazardous-chemical/priority-chemicals/crystalline-silica
SafeWork Qld	https://www.worksafe.qld.gov.au/silicosis/background-to-silicosis
Thoracic Society of Australia and New Zealand	https://www.thoracic.org.au/respiratorylaboratoryaccreditation/spirometry-standards
TSANZ	https://www.thoracic.org.au/documents/item/407
WorkCover WA	https://www.workcover.wa.gov.au/workers/silicosis-claims/
WorkSafe ACT	https://www.accesscanberra.act.gov.au/app/answers/detail/a_id/4646/~/silica-dust
WorkSafe NZ	https://worksafe.govt.nz/topic-and-industry/dust/silica-dust-in-the-workplace/
WorkSafe Tasmania	https://worksafe.tas.gov.au/silicasafe
WorkSafe Victoria	https://www.worksafe.vic.gov.au/crystalline-silica

## Data Availability

Not applicable.
